# *Candida albicans* and *Staphylococcus aureus* reciprocally promote their virulence factor secretion and pro-inflammatory effects

**DOI:** 10.3389/fcimb.2025.1629373

**Published:** 2025-08-22

**Authors:** Raymond Pasman, Bastiaan P. Krom, Martijs J. Jonker, Wim C. de Leeuw, Gertjan Kramer, Stanley Brul, Sebastian A. J. Zaat, Jianbo Zhang

**Affiliations:** ^1^ Department of Molecular Biology and Microbial Food Safety, Swammerdam Institute for Life Sciences, University of Amsterdam, Amsterdam, Netherlands; ^2^ Department of Preventive Dentistry, Academic Centre for Dentistry Amsterdam (ACTA), University of Amsterdam and Free University Amsterdam, Amsterdam, Netherlands; ^3^ RNA Biology Research Group, Swammerdam Institute for Life Sciences, University of Amsterdam, Amsterdam, Netherlands; ^4^ Laboratory for Mass Spectrometry of Biomolecules, Swammerdam Institute for Life Sciences, University of Amsterdam, Amsterdam, Netherlands; ^5^ Department of Medical Microbiology and Infection Prevention, Amsterdam Institute for Immunology and Infectious Diseases, Amsterdam University Medical Centers, University of Amsterdam, Amsterdam, Netherlands; ^6^ Tytgat Institute for Liver and Intestinal Research, Amsterdam Gastroenterology, Endocrinology and Metabolism, Amsterdam University Medical Centers, Amsterdam, Netherlands

**Keywords:** *Candida albicans* (*C. albicans*), *Staphylococcus aureus*, inflammation, virulence factors, macrophages, extracellular virulence factors, nonextracellular virulence factors

## Abstract

**Background:**

Co-infections of *Candida albicans* and *Staphylococcus aureus* can significantly increase morbidity and mortality. However, the effect of *C. albicans–S. aureus* co-existence on virulence factor secretion and pro-inflammatory effects remain elusive.

**Methods:**

We systematically investigated the virulence factors released by *C. albicans* and *S. aureus* under different culturing conditions using proteomics. We characterized their pro-inflammatory effects in macrophages with transcriptomics and gene set enrichment analysis.

**Results and Discussion:**

We showed that co-culturing of *C. albicans* and *S. aureus* promoted the secretion of 7 cytolytic, 11 proteolytic, and 3 lipolytic extracellular virulence factors (ECVFs) and impacted non-ECVFs, owing to Als1/Als3-mediated interactions, the presence of *C. albicans*, or its pH maintenance. Co-culturing promotes *C. albicans* hypha formation and β-glucan masking, suggesting that co-culturing enhances both *C. albicans* invasion and immune evasion. Moreover, the secretome of *C. albicans–S. aureus* co-culture increased pro-inflammatory pathways including promoting TNF-, NFKB-, and Toll-like receptor signaling pathways, as well as cytokine–cytokine receptor interactions in macrophages. Our findings support that *C. albicans* and *S. aureus* reciprocally promote their virulence potential and pro-inflammatory effects, which may provide mechanistic insights into the increased morbidity and mortality during their co-infection *in vivo*.

## Graphical Abstract

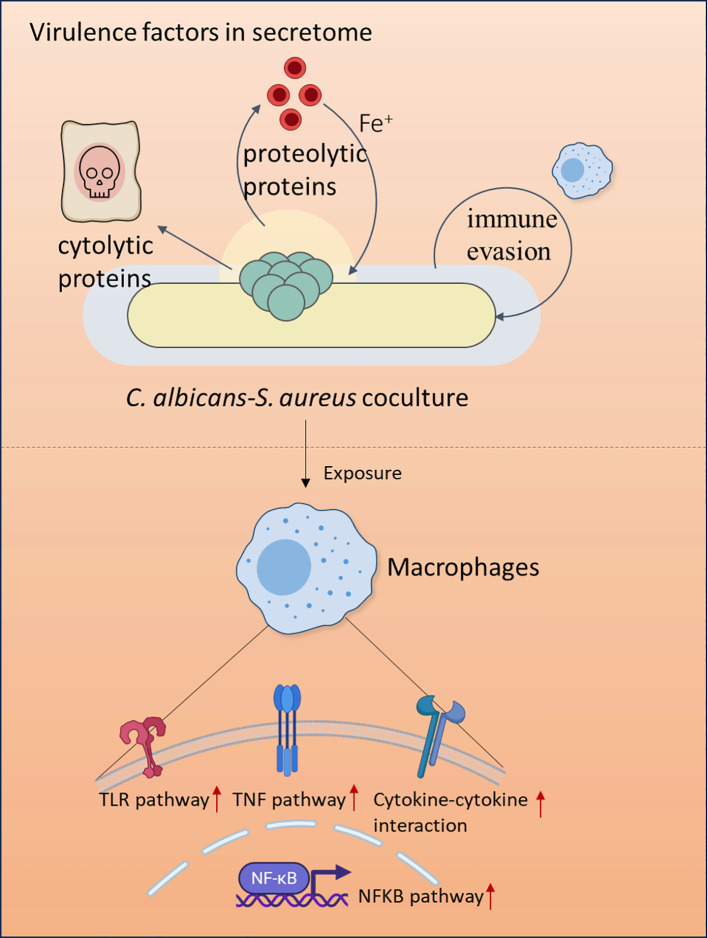

## Introduction

*Candida albicans* is a commensal polymorphic fungus of the human oral, urogenital, gastrointestinal, and skin mycobiome (Berman, 2012; Mayer et al., 2013). In immunocompromised individuals, however, the fungus frequently becomes pathogenic (Pasman et al., 2022). Pathogenicity of *C. albicans* is related to its ability to switch from non-invasive yeast growth to invasive hyphal growth (Talapko et al., 2021). Tissue invasion by hyphae comprises two key processes: the secretion of extracellular virulence factors (ECVFs) that damage the epithelium, facilitating tissue invasion, and invasion by physically forcing the hyphae through epithelial layers (Srikantha et al., 1998; Crampin et al., 2005; Moyes et al., 2016; Desai, 2018). *C. albicans* hyphal invasion can result in a candidal bloodstream infection (BSI) (McCarty et al., 2021). Interestingly, *C. albicans* BSIs frequently co-occur with bacterial bloodstream invasion (Klotz et al., 2007), suggesting that the fungus facilitates co-invasion of bacteria. *Staphylococcus aureus*, an ESKAPE (group of six clinically relevant, highly virulent, and antibiotic-resistant bacterial pathogens) pathogen, is the third most commonly co-isolated bacterium during *C. albicans* BSIs (Klotz et al., 2007) and is the most common pathogen causing primary bacterial BSIs, i.e., BSIs without an identified portal of entry or associated site of infection (del Rio et al., 2009; Pien et al., 2010). Due to their co-isolation during candidal BSIs, *C. albicans* has been hypothesized to account for this lacking staphylococcal portal of entry (Schlecht et al., 2015; Allison et al., 2019).

Various *in vivo* murine studies, using an oral infection model, have shown that *C. albicans* potently promotes co-invasion and dissemination of *S. aureus* and significantly increases lethality compared with monomicrobial infection (Carlson, 1982; Carlson, 1983; Carlson, 1988; Nash et al., 2014; Kong et al., 2015; Schlecht et al., 2015; Nash et al., 2016; Allison et al., 2019; Van Dyck et al., 2021). The *C. albicans* hyphal agglutinin sequence proteins 1 and 3 (Als1, Als3), the proteins responsible for *S. aureus* binding to hyphae, crucially contribute to *S. aureus* co-invasion (Kong et al., 2015; Schlecht et al., 2015; Van Dyck et al., 2021). Furthermore, the *C. albicans* ECVF candidalysin has been shown to significantly contribute to pathogenesis of *C. albicans/S. aureus* co-infections (Van Dyck et al., 2021). Additional *in vitro* studies have shown that *C. albicans/S. aureus* co-culturing also increases the alpha toxin production of the *S. aureus* secretome by promoting the staphylococcal *agr* quorum sensing system in a pH-dependent manner (de Carvalho Dias et al., 2017; Todd et al., 2019b; Todd et al., 2019a; Dias et al., 2021). Aside from candidalysin and staphylococcal alpha toxin, *C. albicans* and *S. aureus* secrete additional damaging ECVFs (cytolytic, proteolytic, and lipolytic), which could potentially contribute to invasion. Furthermore, non-damaging ECVFs can also indirectly increase pathogenicity of both organisms during co-infection by aiding in immune evasion, adhesion, cell wall biosynthesis, and iron acquisition.

*In vivo* murine studies using oral co-infection of *C. albicans* and *S. aureus* have shown that macrophages and neutrophils, isolated from draining oral lymph nodes, contained viable *S. aureus* following co-infection with *C. albicans* but not following any monoculture infections (Allison et al., 2019). Moreover, while low-level immunosuppression crucially contributes to the instigation of invasive candidiasis and the co-invasion/dissemination of *S. aureus* in mice, high-level immunosuppression significantly reduced *S. aureus* dissemination during *C. albicans* co-infection (Van Dyck et al., 2021). This reduced level of *S. aureus* dissemination was linked to both neutropenia and decreased monocyte production which, together, result in a significantly lower number of neutrophils and macrophages at the site of infection in tissue. *In vivo* murine studies using intra-abdominal co-infection of *C. albicans* and *S. aureus* have shown that co-infection amplified host inflammation, resulting in both a significant increase in neutrophil influx towards the site of infection and prolonged neutrophil residence at the site of infection (Peters and Noverra, 2013; Nash et al., 2014). Together, these studies have highlighted the importance of the immune system in the lethal dissemination of *S. aureus* during co-infection with *C. albicans*. The most crucial aspect of immune facilitated dissemination, however, is the fact that *S. aureus* is notorious for surviving phagocytic killing, allowing *S. aureus* to disseminate following phagocytosis and phagocyte migration (Kubica et al., 2008; DuMont et al., 2013; Flannagan et al., 2016; Pidwill et al., 2021). While the secretome of *C. albicans* and *S. aureus* has been shown to promote the murine macrophage production and secretion of IL-6, NO, and TNF-α during co-culturing (de Carvalho Dias et al., 2017), the effect of *C. albicans/S. aureus* co-culture secretomes on human macrophages remains unknown. In addition, the impact of co-culturing on the ECVFs in the secretome remain to be elucidated. Therefore, the aim of this study was to identify which ECVFs are secreted by *C. albicans* and *S. aureus* and how co-culturing influences the virulence potential. We investigated the contribution of Als1/Als3 binding, biofilm integration, and *C. albicans* pH maintenance in mediating the changes in ECVF during co-culturing. Additionally, we tested whether co-culturing of *C. albicans* and *S. aureus* promoted ECVF cytotoxicity towards human oral squamous cells and inflammation in human macrophages.

## Methods

### Strains and growth conditions

*S. aureus* ATCC12600, *C. albicans* SC5314 wild type, and *C. albicans* SC5314 *als1/als3* (genotype: *als1-1Δ*::*FRT/als1-2Δ*::*FRTals3-1Δ*::FRT/*als3-2Δ*::*FRT*) (Van Dyck et al., 2021) were grown as described before (Pasman et al., 2024). In short, *C. albicans* and *S. aureus* strains were maintained from glycerol freezer stocks on Sabouraud dextrose/glucose agar supplemented with chloramphenicol (Sigma, 63567) and mannitol salt phenol red agar (MSA, Sigma, 89579), respectively. Single colonies were added to tryptic soy broth (TSB; Brunschwig Chemie, 211825) and cultured overnight at 37°C, 200 rpm. Cultures were rinsed with Dulbecco’s phosphate-buffered saline (DPBS; 137 mM NaCl, 2.7 mM KCl, 10 mM Na_2_HPO_4_, 1.8 mM KH_2_HPO_4_) and diluted to ~2 × 10^6^ CFU/mL for *C. albicans* and ~2 × 10^7^ CFU/mL for *S. aureus* in Dulbecco’s Modified Eagle’s Medium (Sigma D5030) supplemented with 2.5 g/L dextrose, 1× GlutaMAX (Gibco 35050061), and 1× MEM non-essential amino acids (Gibco 11140050), with a final pH of 7.3 (mDMEM-DMP) (Berman, 2012). Monocultures were generated by further diluting the culture in a 1:1 ratio with mDMEM-DMP, whereas co-cultures were constituted by combining (undiluted) monocultures in a 1:1 ratio. For buffered growth, mDMEM-DMP was supplemented with 100 mM of 4-(2-hydroxyethyl)-1-piperazineethanesulfonic acid (HEPES, Gibco 15630056).

### Biofilm growth and assessment

Mono- and co-culture biofilms were grown by inoculating wells of a six-well plate (Corning 3506) with 3 mL of culture, prepared as described above, and incubating it stationarily for 2 h at 37°C. Each well was washed for three times using DPBS to remove non-adherent cells. Finally, fresh mDMEM-DMP was added to the wells and all plates were incubated stationary for 72 h at 37°C in a humidified environment. Concerning Transwell co-cultures, 1.5 mL of *C. albicans* monoculture was added to the inside of the 0.4 µm Transwell inserts (Thermo Scientific, 140660) whereas 1.5 mL of *S. aureus* monoculture was added to the inside of the well plate. Transwell co-cultures were further treated identical to normal cultures with half the volume added on either side of the membrane. Medium pH was measured by pH 2.0–9.0 strips (Supelco, 1.09584) inoculated with 40 µL of medium. Culture medium was sampled for downstream analysis, as described below. Biofilms were collected using cell scrapers (Greiner, 391-3010), pelleted, and stored at −80°C until further analysis using qPCR.

### Genomic DNA extraction and qPCR

Genomic DNA was extracted from the collected cell pellets using a DNeasy PowerBiofilm Kit (Qiagen, 24000) according to the manufacturer’s protocol. To determine the amount of fungal and bacterial DNA, a quantitative polymerase chain reaction (qPCR) was performed using primers specific to the fungal 28S rRNA gene (forward: GCATATCAATAAGCGGAGGAA AAG; reverse: TTAGCTTTAGATGATTTACCACC; probe: 6FAM-CGGCGAGTG AAGCGGSAARAGCTC-BHQ) (Vollmer et al., 2008) and bacterial 16S rRNA gene (forward: TCCTACGGGAGGCAGCAGT; reverse: GGACTACCAGGGTATCTAATCCTGTT; probe: 6FAM-CGTATTACCGCGGCTGCTGGCAC-BHQ1) (Ciric et al., 2010). Fluorescence was measured using a LightCycler 480 System (Roche) and analyzed using the corresponding system software.

### Secretome sample preparation for proteomic analysis, cytotoxicity assay, and macrophage exposure experiments

To concentrate the secreted proteins in the spent media, the medium collected from six wells of the same culturing conditions was pooled and filtered using a 0.2-µm polyethersulfone (PES) filter (Sarstedt, 83.1826.001). The filtrate was further concentrated with a 3-kDa Amicon ultra centrifugal filter (Pall, MAP003C37). The concentrated secretome samples were immediately stored without protease inhibitor supplementation for cytotoxicity assay, or supplemented with protease inhibitors (Roche, 11873580001) according to manufacture manual for proteomic analysis, or supplemented with RPMI 1640 to the original volume before protein extraction for macrophage activation experiments described below. Protein concentrations were determined using a Bradford protein assay, and prior to LC-MS proteomic analysis, protein concentrations were diluted to identical levels across all samples. All secretome samples were stored at −80°C until use.

### Sample preparation for LC-MS analysis

Samples were prepared and measured according to Šimkovicová et al. (2024) (Šimkovicová et al., 2024). In short, samples were thawed, reduced, and alkylated by incubation with tris-(2-carboxyethyl)phosphine (10 mM) and chloroacetamide (30 mM) for 30 min, 70°C. Next, samples were prepared for mass spectrometry analysis using the single-pot, solid-phase-enhanced sample preparation (SP3) protocol (Hughes et al., 2014). Soluble protein recovery was optimized by ensuring no detergents were added to the samples and precipitation time was extended to 30 min (room temperature). Beads used for washing were air-dried and resuspended in ammonium bicarbonate (100 mM) after which trypsin (Sequencing Grade Modified) was added at a protease-to-protein ratio of 1:50 (w/w) at 37°C. Formic acid was added to the overnight digestion at a final concentration of 1% and pH of 2. Finally, peptides were recovered using a magnetic separator device.

### LC-MS analysis for quantitative proteomics

Samples were separated by reversed-phase chromatography using an UltiMate 3000 RSLCnano UHPLC system (Thermo Scientific, Germeringen, Germany), and peptides were separated using a 75 μm × 250 mm analytical column (C18, 1.6 μm particle size, Aurora, IonOpticks, Australia), maintained at 50°C, and operated at a flow rate of 400 nL/min with 3% solvent B for 3 min (solvent A: 0.1% formic acid in water, solvent B: 0.1% formic acid in acetonitrile, ULCMS-grade, Biosolve). Next, a multi-stage gradient was applied (17% solvent B at 21 min, 25% solvent B at 29 min, 34% solvent B at 32 min, 99% solvent B at 33 min, kept at 99% solvent B till 40 min). For equilibration, the system was returned to initial conditions (t = 40.1 min) for 58 min. Eluted peptides were electrosprayed by a captive spray source via the column-associated emitter and were analysed by a TIMS-TOF Pro mass spectrometer (Bruker, Bremen, Germany) operated in PASEF mode for standard proteomics acquisition. MS/MS scans were initiated 10× with a total cycle time of 1.16 s, a target intensity of 2 × 10^4^, an intensity threshold of 2.5 × 10^3^, and a charge state range of 0–5. Active exclusion was enabled for a period of 0.4 min and precursors reevaluated when the ratio of current intensity:previous intensity exceeded 4.

### Spectral data processing and proteome database search

LC-MS data were processed using MaxQuant software (version 1.16.14.0) using standard settings, i.e., trypsin/p as the enzyme allowing for two missed cleavages with carbamidomethylation at cysteine as a fixed modification and oxidation at methionine as a variable modification searching the proteome databases of: Candida_albicans_UP000000559 and Saureus_UP000008816. MaxQuant outputs were used for subsequent analysis using Perseus (version 2.0.7.0). Proteins that are only identified by peptides carrying one or more modified amino acids as well as reverse and potential contaminant proteins were removed from the dataset. Remaining data were log2 transformed, and proteins that were not measured in both samples of at least one condition were removed. Next, remaining proteins were annotated using the 2019_11 release of the Max Planck Institute of Biochemistry annotation database (*C. albicans* SC5314 and *S. aureus* NCTC 8325), after which the dataset was split into two sets: one set containing all *C. albicans* proteins and one set containing all *S. aureus* proteins. For both sets, missing values were imputed based on the low end of the corresponding normal distribution (width = 0.3, down shift = 1.8) and all values were subtracted with the most frequent value. Protein differences were tested on significance using ANOVA with a permutation-based FDR of 0.05 and 250 number of randomizations after which non-significant proteins were removed. Principal component analysis was used to identify sample group differences based on the remaining proteins. Finally, remaining data were normalized using Z-score normalization and significantly differing proteins between monoculture and co-culture conditions were identified using volcano plots based on a Pearson correlation with an FDR of 0.05. Using the virulence factor database (Liu et al., 2022), Aureo Wiki (Fuchs et al., 2018), UniProt (UniProt Consortium, 2018), STRING database (Szklarczyk et al., 2023), and various studies (Calderone and Fonzi, 2001; Sorgo et al., 2010; Zecconi and Scali, 2013), extracellular (ECVFs) and non-extracellular (N-ECVFs) virulence factors were identified. Results were visualized using Microsoft Excel (version 2402, build 17328.20282).

### Human oral squamous cell culture and cytotoxicity assay

Ca9-22 (gingival squamous carcinoma) and HO1N1 (buccal epithelial carcinoma) cells were cultured in DMEM + 10% foetal bovine serum (FBS; Sigma) at 37°C, 5% CO_2_. Cells reached confluence within 10 days and were washed using PBS, detached by trypsinization (0.05% trypsin) for 3 min at 37°C, spun down, and diluted to 1 × 10^5^ cells/mL using DMEM/F12 (Gibco) supplemented with 10% FBS. Cells were inoculated (1 × 10^5^ cells/well) into wells of a 24-well plate (Greiner) and incubated for 24 h at 37°C, 5% CO_2_. Ca 9–22 and HO1N1 cells were exposed to secretome protein isolates (1:10 diluted in mDMEM-DMP, 100 mM HEPES), acquired as described above but without protease inhibitor supplementation, and incubated for 24 h at 37°C, 5% CO_2_. Medium was collected and stored at −20°C until cytotoxicity assay. Cytotoxicity was determined using a Roche LDH Kit PLUS (Cat. No. 04 744 934 001) according to the manufacturer’s protocol. Medium of cells lysed with 1% Triton-X 100 in mDMEM-DMP (100 mM HEPES) was used as positive control, whereas medium of unexposed macrophages was utilized as negative control.

### THP-1 cell culture and differentiation

THP-1 monocytes were maintained in culture in Roswell Park Memorial Institute medium (RPMI 1640, Gibco, 11875093) supplemented with 10% foetal bovine serum (Sigma F9665), 1% GlutaMAX (Gibco, 35050061), and 1% Pen-strep (Gibco, 15140-122). THP-1 monocytes were seeded in either 96- or 24-well plates at a density of 0.5 × 10^6^ cells/mL and differentiated into M0 macrophages by incubating the cells for 72 h at 37°C, 5% CO_2_ in RPMI 1640 supplemented with 100 nM phorbol 12-myristate 13-acetate (PMA, Millipore 500582). Following a 72-h incubation, cells were washed with Dulbecco’s phosphate-buffered saline (DPBS, Gibco, 14190177, 37°C) and used for downstream application. Differentiated macrophages were imaged at 10× magnification using a Leica DM13000 B coupled to a ZEISS Axiocam MRc.

### Secretome cytotoxicity

THP-1 M0 macrophages, differentiated in 96-well plates, were incubated in RPMI 1640 supplemented with various dilutions of the conditioned media (1:25, 1:50, 1:100, and 1:200, diluted in RPMI 1640) for 6 h at 37°C, 5% CO_2_. Macrophages in RPMI 1640 served as the negative control, whereas macrophages treated with 1% Triton-X 100 for 10 min before medium collection served as the positive control. Following incubation, medium was collected and LDH activity was quantified using an LDH-Glo™ Cytotoxicity Assay (Promega, J2380), measured with a Synergy Mx (Bio Tek). Cytotoxicity was determined as follows: Percentage of cytotoxicity (%) = 100 * ((Experimental LDH release – negative control)/(Positive control LDH release – negative control)).

### RNA isolation, sequencing, and data analysis

Macrophages were lysed by incubating in 350 µL of 1% Triton-X 100 (Bio-Rad) in DPBS for 10 min. Subsequently, 350 µL of buffer RLT in the RNeasy Plus Mini Kit (Qiagen, 74134) supplemented with 1% β-mercaptoethanol was added, mixed with pipetting, harvested in 1.5-mL tubes, and stored at −70°C until RNA isolation. The total RNA was then isolated by following the manufacturer’s protocol. The RNA purity was measured using a DeNovix DS-11+ spectrophotometer, with RNA integrity number of all samples 9.9–10.0 except for one at 9.7 due to the high concentration of total RNA.

The mRNA enrichment and library preparation was carried out by the Dutch Genomics Service & Support Provider at the University of Amsterdam. The NEBNext Poly(A) mRNA Magnetic Isolation Module (New England Biolabs) was used to perform a poly-A enrichment using 1 μg total RNA. RNA-Seq libraries were generated according to the manufacturers’ protocols using the NEBNext Ultra II Directional RNA Library Prep Kit for Illumina and NEBNext Multiplex Oligos for Illumina (Unique Dual Index Primer Pairs) (New England Biolabs). The size distribution of the libraries with indexed adapters was assessed using a 2200 TapeStation System with Agilent D1000 ScreenTapes (Agilent Technologies). The libraries were quantified on a QuantStudio 3 Real-Time PCR System (Thermo Fisher Scientific) using the NEBNext Library Quant Kit for Illumina (New England Biolabs) according to the instructions of the manufacturer. The libraries were clustered and sequenced (75 bp) on a NextSeq 550 System (Illumina) using a NextSeq 500/550 High Output Kit v2.5 (75 Cycles) (Illumina).

The raw sequencing data were subjected to quality control with FastQC v0.11.9 (Andrews, 2010) and MultiQC version 1.21 (Ewels et al., 2016). Subsequently, the reads were trimmed with Trimmomatic v0.39 (Bolger et al., 2014). Post-trimming quality control indicated that all samples were of similar high quality. The reads were aligned (without soft clipping) to the human reference genome (GRCh38.111) using HISAT2 version 2.2.1 (Kim et al., 2015). HTSeq-count version 2.0.5 (Anders et al., 2015) was used to count the amount of reads per gene and determine the gene expression values. The gene expression data were checked for quality and subsequently normalized and analysed with DESeq2 (Love et al., 2014). The technical replicates were collapsed and statistical tests controlling for batch effects were performed. The differences between the monocyte control and the medium control were assessed by comparing differentially expressed genes (DEGs), i.e., genes with a FDR-adjusted p-value < 0.01 and a log2 fold change above 1 or below −1, with previously identified DEGs following THP-1 monocyte differentiation (Liu et al., 2023). DEGs were identified between all conditions and visualized using Microsoft Excel (version 2403). Expression of M1 and M2 polarization related genes was identified according to previously published gene sets for differential gene expression (Mills and Ley, 2014; Murray, 2017; Shapouri-Moghaddam et al., 2018; Rynikova et al., 2023). Finally, to identify pathways containing overrepresented genes, a Gene Set Enrichment Analysis (GSEA) was performed using WebGestalt 2019 (Liao et al., 2019) with a KEGG-based pathway analysis, a weighted set cover redundancy removal, and a significance level cutoff of FDR-adjusted p-value < 0.01. WebGestalt output pathways were visualized using both Microsoft Excel (version 2403) and Python (version 3.8.5). Gene ratios were determined by dividing the number of enriched genes in a pathway by the total number of genes of that pathway.

### Statistical analysis

All data were tabulated and visualized using Microsoft Excel (version 2403). When applied, data normality was tested using a Shapiro–Wilk test, and group comparison was performed using either a one-way ANOVA (normally distributed data) or Kruskal–Wallis test (non-normally distributed data) combined with a Tukey post analysis using Prism graph pad (8.3.0). All conditions were tested in at least three biological replicates and technical duplicates.

## Results

### Co-culturing of *C. albicans* and *S. aureus* increases the ECVF and N-ECVF secretion by *C. albicans*

**Figure 1:**
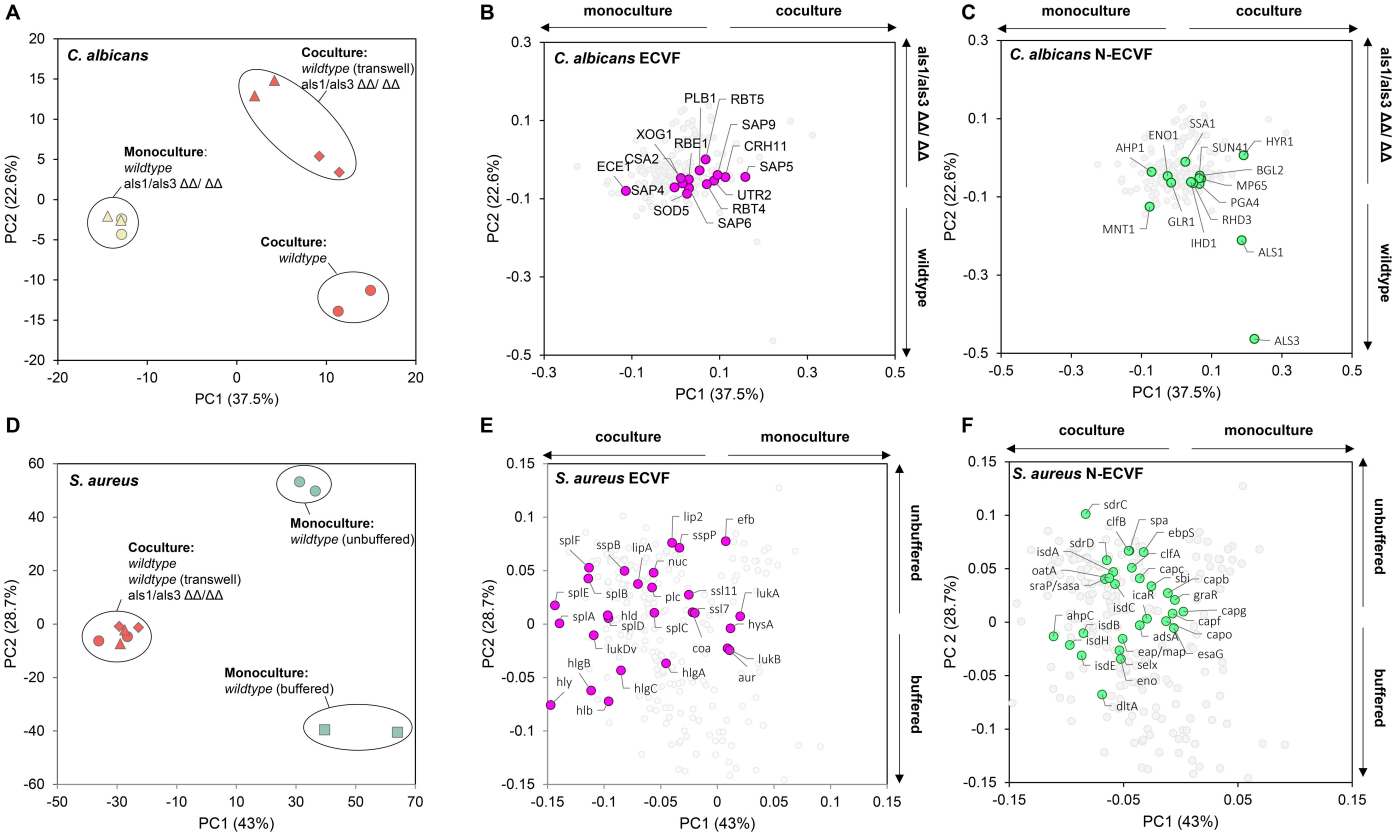
Co-culturing changes extracellular virulence factors and non-extracellular virulence factors released by *C. albicans* and *S. aureus*. **(A)** Principal component analysis and **(B, C)** corresponding loading plot of *Candida albicans* spent medium proteins that were detectable in at least one of the samples. Extracellular virulence factors (ECVF) and non-ECVF (N-ECVF) are highlighted in magenta in **(B)** and in green in **(C)**, respectively. Culture conditions in **(A)** yellow filled circle: *C. albicans* wild-type, yellow filled triangle: *C. albicans ALS1/ALS3* ΔΔ/ΔΔ, red filled circle: co-culture wild-type, red filled triangle: co-culture *ALS1/ALS3* ΔΔ/ΔΔ, red filled diamond: co-culture Transwell. *C. albicans* ECVFs are colored in magenta in **(B)**. **(D)** Principal component analysis and **(E, F)** corresponding loading plot of *Staphylococcus aureus* spent medium proteins that were detectable in at least one of the samples. ECVF and N-ECVF are highlighted in magenta in **(E)** and in green in **(F)**, respectively. Culture conditions in **(C)**: red filled circle: co-culture wild-type, red filled triangle: co-culture *ALS1/ALS3* ΔΔ/ΔΔ, red filled diamond: co-culture Transwell, green filled square: *S. aureus* monoculture buffered, green filled circle: *S. aureus monoculture* unbuffered. *S. aureus* ECVFs are colored in pink in **(E)**.

To investigate the effect of *C. albicans* and *S. aureus* co-culturing on the secreted proteins, we measured the levels of the proteins in the spent media collected from different co-culture and monoculture conditions (see Methods). Following mass spectrometry analysis, we detected 183 C*. albicans* proteins in both samples of at least one culturing condition. Principal component analysis (PCA) revealed a clear separation between co-cultures and monocultures (**Figure 1A**), indicating that *C. albicans-*secreted protein profiles were changed by the presence of *S. aureus*. Of the 21 known *C. albicans* ECVFs (**Supplementary Table S1**), 14 ECVFs were detected in both samples of at least one culturing condition and 12 ECVFs were significantly increased in co-culture compared with monoculture (**Figure 1B**; **Supplementary Table S1**). Among these, five ECVFs, i.e., Plb1, Sap4, Sap6, Sod5, and Csa2, were significantly increased only in wild-type co-culture compared with monoculture, but this increase was abolished in *ALS1/ALS3* ΔΔ/ΔΔ or Transwell setting (**Supplementary Table S1**), suggesting that their secretion was strongly dependent on Als1/Als3-mediated binding, whereas secreted aspartic protease 5 (Sap5) and glycosidase Crh11 was independent of Als1/Als3-mediated binding and other types of physical interaction (**Supplementary Table S1**). Interestingly, the remaining five ECVFs, i.e., Sap9, glycosidase Utr2, glucan 1,3-beta-glucosidase Xog1, PR-1 protein homolog Rbe1, and Rbt4, were significantly increased during both regular and Transwell-separated co-culturing with wild-type *C. albicans* but were not changed following co-culturing with *C. albicans ALS1*/*ALS*3 ΔΔ/ΔΔ (**Supplementary Table S1**), indicating that their release was strongly dependent on soluble factors and independent of Als1/Als3-mediated binding (**Figure 1B**; **Supplementary Table S1**). These significantly increased *C. albicans* ECVFs contribute to damaging functions such as proteolysis, immune evasion, cell wall modelling, and iron acquisition, suggesting that co-culturing *C. albicans* with *S. aureus* promotes virulence of *C. albicans*. It is worth noting that *C. albicans* candidalysin, a known virulence factor, does not contribute to the virulence increase in co-cultures since *C. albicans* candidalysin was detected but did not significantly change following co-culturing with *S. aureus* (**Supplementary Table S1**).

Next, we expanded our investigation to non-extracellular virulence factors (N-ECVFs), as cytoplasmic proteins in *C. albicans* were showed to be released extracellularly via extracellular vescicles (Gil-Bona et al., 2015). We identified 42 N-ECVFs, of which 14 C*. albicans* N-ECVFs were significantly changed and mainly contributed to co-culture-based (PC1, 37.5%) and *ALS1/ALS3* deletion-based (PC2, 22.6%) separation (**Figure 1C**). Most of the 14 significantly changed *C. albicans* N-ECVFs were related to adhesion or cell wall remodeling. For instance, hyphae-regulated cell wall protein 1 (Hyr1) and heat shock protein 70 (Ssa1) were significantly increased in wild-type co-culture versus *C. albicans* monoculture, but this change was attenuated in als1/als3 ΔΔ/ΔΔ co-culture and Transwell settings (**Supplementary Table S2**). Additionally, thioredoxin peroxidase (Ahp1) and glycolipid 2-alpha-mannosyltransferase 1 (Mnt1), involved in the reduction of hydrogen peroxide and O-glycosylation of cell wall, respectively, were significantly decreased in wild-type and Transwell co-culture, and this decrease was enhanced in *ALS1/ALS3* ΔΔ/ΔΔ co-culture. These results suggest that Hyr1, Ssa1, Ahp1, and Mnt1 are influenced by both Als1/Als3-dependent *C. albicans–S. aureus* interaction and other *S. aureus* factors. In addition, cell wall protein (Rhd3) and cell wall remodeling-related proteins Ihd1, Sun41, Pga4, Als1, Als3, Mp65, and Bgl2 were significantly increased in wild-type co-culture (**Supplementary Table S2**), which was attenuated by the physical separation and completely abolished in *ALS1/ALS3* ΔΔ/ΔΔ co-culture (**Supplementary Table S2**). Moreover, the enolase (Eno1) level was not altered during wild-type co-culturing, whereas its level was decreased in *ALS1/ALS3* ΔΔ/ΔΔ and Transwell co-cultures (**Supplementary Table S2**). Together, these results suggest that the Als1/Als3-dependent and independent physical interaction between *C. albicans* and *S. aureus* promotes the levels of non-extracellular virulence factors.

### Co-culturing of *C. albicans* and *S. aureus* promotes ECVF and N-ECVF secretion by *S. aureus*

Co-culturing also influenced the release of virulence factors by *S. aureus*. We detected 930 *S. aureus* proteins in at least one culturing condition. Principal component analysis indicates a clear separation between monocultures and co-cultures (**Figure 1D**), suggesting that the presence of *C. albicans* has substantial impact on the proteins released by *S. aureus*. It is worth noting that no separation was observed among three co-culture conditions (**Figure 1D**), indicating that deletion of *ALS1/ALS3* or lack of physical interaction with *C. albicans* did not influence the *S. aureus* secretome compared with wild-type co-culture. Of the 50 known *S. aureus* ECVFs, 27 were detected in both samples of at least one culture condition and 20 were found statistically significantly changed (**Figure 1E**, **Supplementary Table S3**). Of the 20 changed ECVFs, hemolysins including alpha hemolysin (Hly/Hla), Hlb, and HlgA-C were increased in all conditions (**Supplementary Table S3**). During the co-culture, *C. albicans* can maintain the pH (**Supplementary Figure S1A**) from decreasing caused by *S. aureus*. Here, we observed that buffering the pH alone increased the secretion of these hemolysins to a similar extent as the *C. albicans*, and this increase was maintained despite the lack of physical contact or Als1/Als3-mediated binding (**Figure 1E**; **Supplementary Table S3**). This result, together with the maintained pH during *C. albicans–S. aureus* co-culture (**Supplementary Figure S1A**), suggests that *C. albicans*-maintained pH likely contributed to the increased release of the hemolysins. On the other hand, other *S. aureus* ECVFs were not influenced by pH but by other factors during co-culturing. For instance, cysteine proteinase staphopain B (SspB), serine protease-like protein A-F (SplA-F), lipase (Lip), phospholipase C (Plc), delta hemolysin (Hld), and leukotoxin D (LukD) were significantly more present in all co-culture conditions, but significantly less present or unaltered during buffered monoculture (**Supplementary Table S3**). Because *ALS1/ALS3* deletion and separated growth did not deviate from wild-type co-culture results, these results are independent of Als1/Als3 and physical binding.

Similar to *C. albicans*, we also detected *S. aureus* N-ECVFs. Of the 28 detected N-ECVFs, 17 were significantly changed (**Figure 1F**). Buffering strongly impacted N-ECVF levels, evidenced by the fact that 11 and 2 N-ECVFs in buffered monoculture were significantly higher and lower than those in unbuffered monoculture, respectively (**Figure 1F**). This pH-mediated effect seemed to be divergently interfered with other factors from *C. albicans*. For example, the buffering increase of d-alanine-d-alanyl carrier protein ligase (DltA) and enolase (Eno) was further enhanced by the presence of *C. albicans*, and this enhancement is independent of Als1/Als3 or physical proximity (**Figure 1F**; **Supplementary Table S4**). For those proteins that were decreased by buffering, i.e., ClfB, Ebp, Oata, Srap/Sasa, IsdA, IsdC, IsdE, SpA, SdrC, and ClfA, the decrease was attenuated or even reserved by the presence of *C. albicans* (**Supplementary Table S4**), despite that *C. albicans* maintained pH comparably with HEPES buffering (**Supplementary Figure S1A**). These results suggest that other *C. albicans*-derived soluble factors have stronger effects in influencing these proteins compared with pH. Together, these results suggest that both *C. albicans*-derived pH maintenance has a broader impact than that of Als1/Als3-mediated binding on N-ECVFs of *S. aureus*.

### Co-culturing promotes cytotoxicity to oral squamous cells

**Figure 2:**
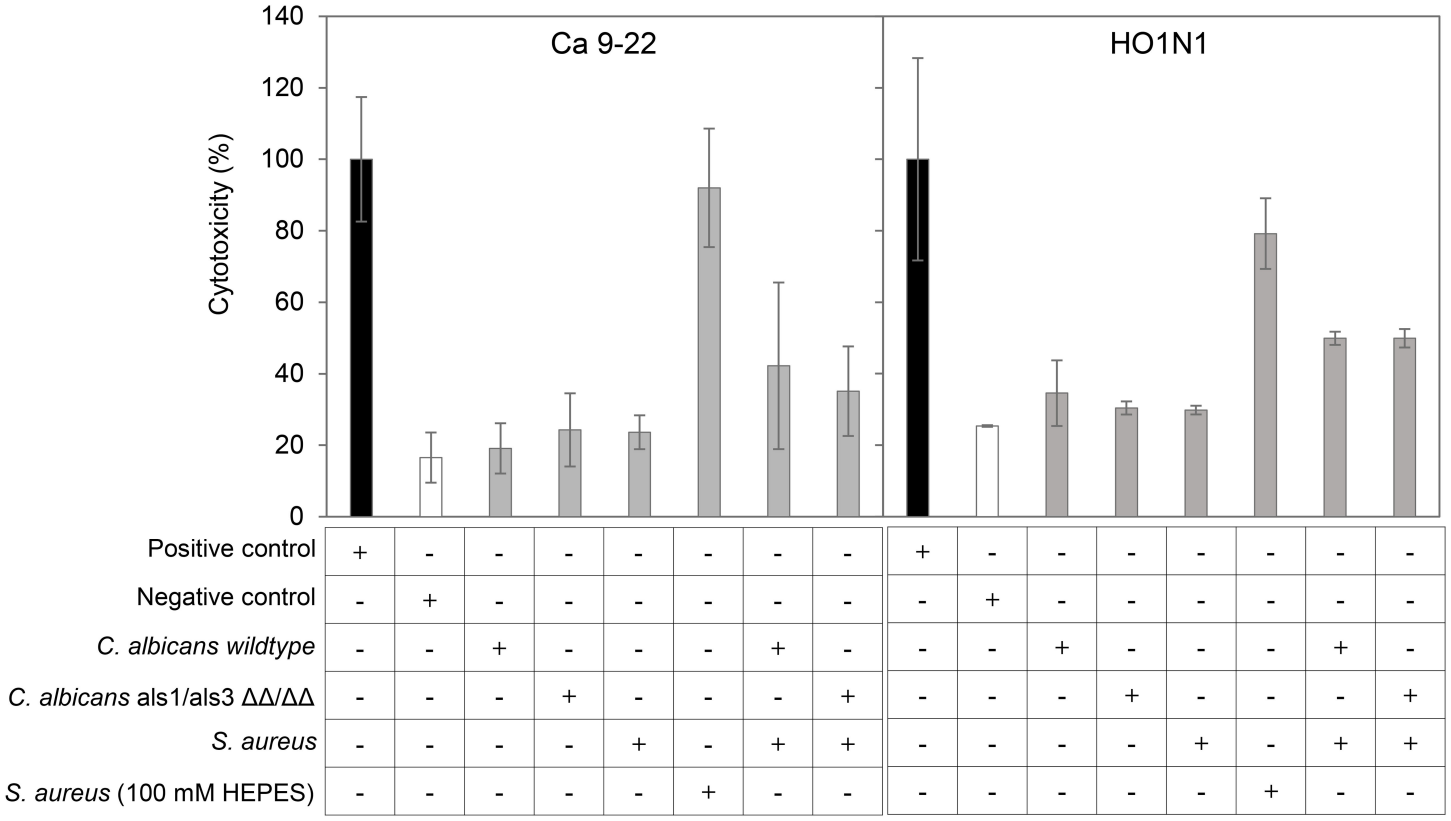
Co-culturing *C. albicans* and *S. aureus* induces slightly higher cytotoxicity on human oral epithelial cells. Cytotoxicity, expressed as the percentage of LDH activity in the medium relative to positive control 1% Triton X-100, after a 24-h exposure to secretomes from different culturing conditions on human gingival/oral squamous carcinoma Ca 9–22 and HO1N1 cells. Error bars represent standard variation of three wells in the same experiment. Negative control: corresponding medium (mDMEM-DMP).

Previous *in vivo* murine studies have reported that oral inoculation of *C. albicans* potently promotes co-invasion and dissemination of co-inoculated *S. aureus* (Carlson, 1982; Carlson, 1983; Carlson, 1988; Nash et al., 2014; Kong et al., 2015; Schlecht et al., 2015; Nash et al., 2016; Allison et al., 2019; Van Dyck et al., 2021), suggesting that epithelium in the oral cavity is disrupted by co-infection. Reasoning that the increase in ECVF and N-ECVF in *C. albicans–S. aureus* co-culture would increase the damaging potential, we next sought to test whether the co-culture induces higher cytotoxicity to oral squamous cells. To test this, we exposed human gingival squamous Ca 9–22 and human buccal mucosa squamous HO1N1 cells to undiluted mono- and co-culture secretomes of *C. albicans* wild-type*, C. albicans ALS1/ALS3* ΔΔ/ΔΔ, and/or *S. aureus* (cultured in either buffered or unbuffered medium) for 24 h and determined cytotoxicity by measuring lactate dehydrogenase activity in the medium. All monoculture secretomes, i.e., *C. albicans* wild-type*, C. albicans ALS1/ALS3* ΔΔ/ΔΔ, and *S. aureus* (unbuffered), induce similar levels of cytotoxicity as the negative control (~20%–30%; **Figure 2**). In contrast, all tested co-culture secretomes and *S. aureus* buffered induced higher cytotoxicity to both Ca 9–22 cells and HO1N1 cells (**Figure 2**). These results are in agreement with the observed elevated levels of ECVFs in co-culture secretomes compared with monoculture. Interestingly, the secretome of buffered *S. aureus* monocultures showed higher cytotoxicity to Ca 9-22 (92%) and HO1N1 (79%) (**Figure 2**), despite that the levels of ECVFs in buffered *S. aureus* were comparable or lower than that in co-cultures (**Supplementary Table S3**).

### The *S. aureus* but not *C. albicans* monoculture secretome is cytotoxic to macrophages and induces inflammation under non-cytotoxic doses

**Figure 3:**
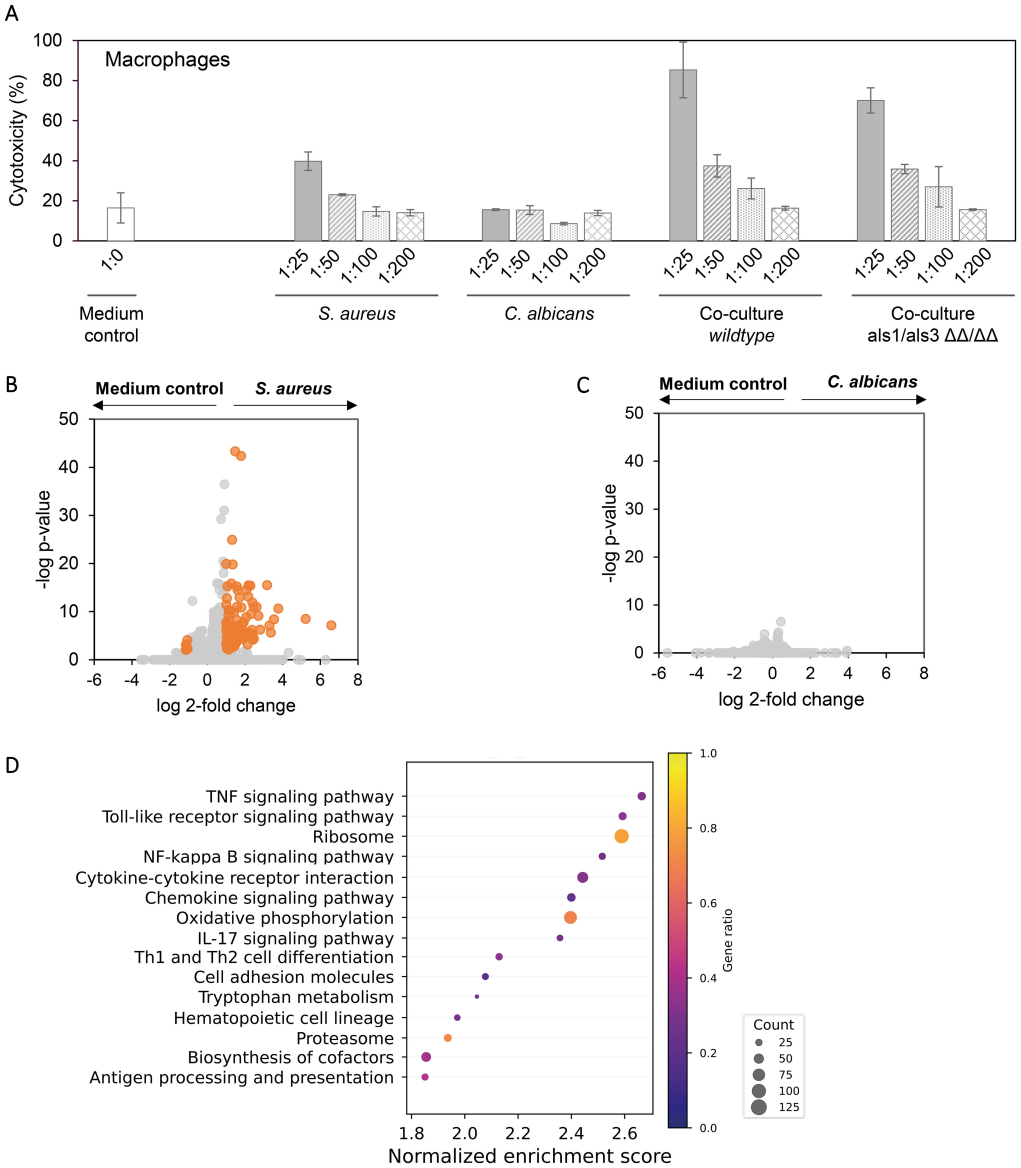
Effects of *S. aureus* and *C. albicans* secretome in macrophages. **(A)** Cytotoxicity of secretome on THP-1-derived macrophages using LDH assay. Secretome with a 1:200 dilution does not induce apparent cytotoxicity as compared with medium control (RPMI). **(B)** Volcano plot of gene transcriptional change in macrophages exposed to *S. aureus* secretome versus no exposure. **(C)** Volcano plot of gene transcriptional change in macrophages exposed to *C. albicans* secretome versus no exposure. Genes with a log2 fold change above 1 or below −1 and an FDR-adjusted p-value < 0.01 were considered significant (orange). **(D)** Gene set enrichment analysis based on the log2 fold change gene expression between macrophages exposed to *S. aureus* secretome and unexposed macrophages. Represented pathways are displayed. Dot sizes represent the total number of enriched genes found in the corresponding pathway, and color represents the gene ratio.

Next, we sought to investigate the effects of co-culturing on macrophages. Macrophages are first-line defense mechanisms during the infection in oral mucosa and are expected to encounter *C. albicans* and *S. aureus* during initial co-invasion of the mucosa. We differentiated THP-1 monocytes into M0 macrophages and verified the differentiation by comparing the macrophage and monocyte transcriptomes, as well as macrophage morphology (**Supplementary Figure S2**). Secretome of monocultures and co-cultures induced different levels of cytotoxicity to these macrophages (**Figure 3A**). Surprisingly, *C. albicans* monoculture showed similar cytotoxicity as the medium control. Similar to oral cells, co-cultures induced higher cytotoxicity than *S. aureus* and *C. albicans* monocultures, and the cytotoxicity is dose-dependent, with 1-to-200 dilution showing the same level of cytotoxicity as the medium (**Figure 3A**). We next sought to identify the molecular response in macrophages that were exposed to a non-cytotoxic level of secretome. Transcriptomic analysis revealed that *S. aureus* alone significantly increased the expression of 93 and decreased 8 (**Figure 3B**), with genes related to M1 macrophage polarization were mostly upregulated (**Supplementary Figure S3**), and M2 polarization-related genes were hardly affected (**Supplementary Figure S4**). Consistently, multiple proinflammatory pathways were increased in *S. aureus* secretome-exposed macrophages (**Figure 3C**). In contrast, *C. albicans* secretome did not significantly change the transcription of any genes in macrophages (**Figure 3D**), which is in agreement with the cytotoxicity data (**Figure 2**).

### Secretome of *C. albicans* and *S. aureus* co-cultures amplifies inflammatory responses of THP-1 macrophages compared with monoculture secretomes

**Figure 4:**
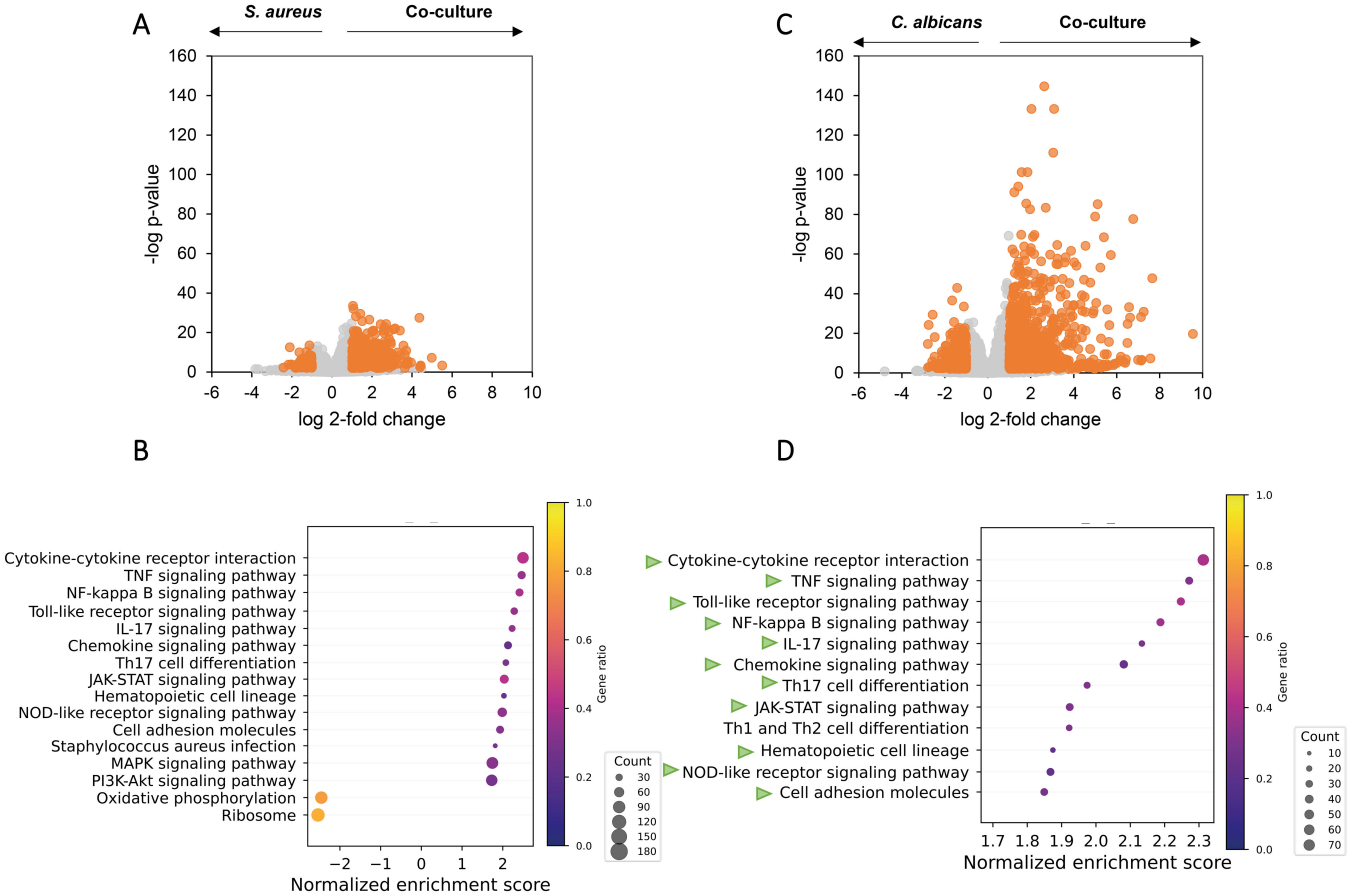
Co-culturing of *C. albicans* and *S. aureus* increases pro-inflammatory pathways in macrophages at the transcriptomic level. Volcano plot and overrepresented pathways on the transcriptomes of the macrophages exposed to secretomes from *C. albicans* wild-type-*S. aureus* co-culture versus secretomes from monoculture of *S. aureus* **(A, B)** or *C. albicans* **(C, D)**. **(A, C)** Genes with a Log2 fold change above 1 or below −1 and an FDR-adjusted p-value < 0.01 were considered statistically significant and are highlighted in orange. **(B, D)** Gene set enrichment analysis based on the Log2 fold change ranking; see more details in Methods. Overrepresented pathways are displayed. Dot sizes represent the total amount of enriched genes found in the corresponding pathway, and color represents the gene ratio. Green arrows in **(D)** represents the same pathways that are overrepresented in **(B)**.

Compared with *S. aureus* monoculture secretome, THP-1 M0 macrophages exposed to co-culture secretome showed 467 significantly upregulated genes and 87 downregulated genes (**Figure 4A**), including higher transcription of genes related to M1 polarization, pro-inflammatory cytokines and chemokines (**Supplementary Figure S3**). Despite the activation of macrophages by *S. aureus* monoculture, 29 pro-inflammatory pathways were further enriched in co-culture secretome-exposed macrophages (**Figure 4B**), with the majority of these pathways (TNF signaling, TLR signaling, NFKB signaling, and cytokine–cytokine receptor interaction) upregulated as a result of exposure to *S. aureus* secretome versus medium. In agreement, co-culture secretome-exposed macrophages showed enrichment of the NOD2-like receptor signaling pathway, a known activating pathway of these pro-inflammatory pathways. Interestingly, pathways related to ribosomal and oxidative phosphorylation proteins were significantly negatively affected by exposure to co-culture secretome, indicating metabolic downregulation. When compared with *C. albicans* monoculture secretome, co-culture secretome-exposed macrophages showed 784 upregulated DEGs and 312 downregulated DEGs (**Figure 4C**) and enriched the proinflammatory pathways that are vastly overlapping with those when co-culture vs. *S. aureus* monoculture (**Figure 4D**).

### *Als1/Als3* deletion has a marginal impact on the proinflammatory effects of co-culture secretome

**Figure 5:**
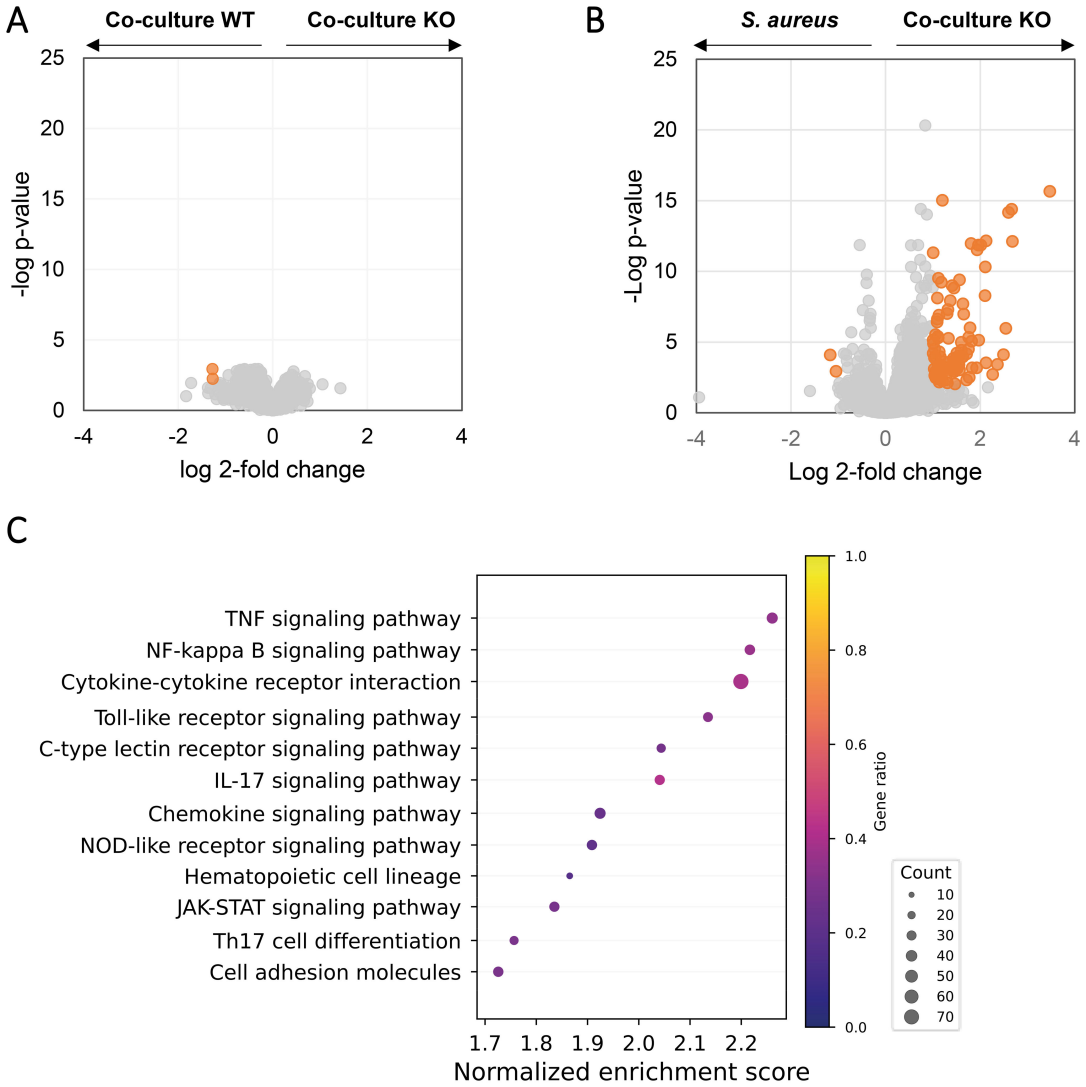
Knockout of als1/als3 in *C. albicans* does not attenuate the pro-inflammatory potential of the *C. albicans*–*S. aureus* co-culture. Volcano plot of gene expression in macrophages exposed to the secretomes from *C. albicans ALS1/ALS3* ΔΔ/ΔΔ–*S. aureus* co-culture versus *C. albicans* wild-type–*S. aureus* co-culture **(A)** or *S. aureus* monoculture **(B)**. Genes with a Log2 fold change above 1 or below −1 as well as an FDR-adjusted p-value < 0.01 were considered statistically significant and highlighted in orange. **(C)** Overrepresented pathways in macrophages exposed to the secretome from *C. albicans ALS1/ALS3* ΔΔ/ΔΔ–*S. aureus* co-culture versus *S. aureus* monoculture based on gene set enrichment analysis. Dot sizes represent the total amount of enriched genes found in the corresponding pathway and color represent the gene ratio.

Reasoning that Als1/Als3 plays an important role in the virulence factor pattern in *C. albicans–S. aureus* co-culture, we hypothesize that depletion of *ALS1/ALS3* will significantly alleviate the proinflammatory effects induced by wild-type *C. albicans–S. aureus* co-culture. To test this hypothesis, we compared the transcriptome of macrophages exposed to the wild-type co-culture secretome versus *C. albicans ALS1/ALS3* ΔΔ/ΔΔ–*S. aureus* co-culture secretome. Surprisingly, only two genes were significantly changed (**Figure 5A**), indicating that *ALS1/ALS3* gene deletion has marginal impact on the gene transcription in macrophages. Compared with *S. aureus* secretome, *ALS1/ALS3* ΔΔ/ΔΔ co-culture secretome showed 97 upregulated genes and 2 downregulated genes in macrophages (**Figure 5B**). Despite that there were less significantly changed genes in *ALS1/ALS3* ΔΔ/ΔΔ co-culture versus *S. aureus* compared with wild-type co-culture versus *S. aureus*, similar proinflammatory pathways (**Figure 5C**), including the TLR2 and NOD2-like receptor signaling pathways, were enriched in *ALS1/ALS3* ΔΔ/ΔΔ co-culture, affirming that *ALS1/ALS3* deletion does not affect the proinflammatory effects of the co-culture.

## Discussion

Previous studies have shown that co-infections of *C. albicans* and *S. aureus* significantly promote lethality compared with mono infections (Carlson, 1988; Nash et al., 2014; Kong et al., 2016; Nash et al., 2016). Co-invasion and dissemination of *S. aureus* are crucial to this process and are facilitated by the secretion of damaging ECVFs and hyphal invasion of *C. albicans*. Our and others’ previous work have shown that *C. albicans* hyphal formation and invasion is increased by soluble factors of *S. aureus* (Peters et al., 2010). Using proteomics, we systemically profiled the ECVFs and N-ECVFs released by both *C. albicans* and *S. aureus* under various *in vivo*-relevant conditions. Our results showed that co-culturing significantly increased the levels of ECVFs and N-ECVFs. While Als1/Als3 binding mainly influenced the *C. albicans* virulence factors, *C. albicans*-mediated pH maintenance primarily contributed to the increase of *S. aureus* virulence factors. The increase of these virulence factors promotes cell cytotoxicity and proinflammatory effects.

### *C. albicans* virulence is promoted by *S. aureus* during co-culturing and mainly attributed to Als binding

ECVFs are essential for various pathogenic processes of *C. albicans*, such as hyphal formation, damaging host cells, invasion, and immune evasion. Previously, Als3, Csa2, Rbt4, Sap4, and Sap6 proteins were found to be enriched in the secretome of N-acetylglucosamine-induced hyphal growth over yeast and contribute to the virulence of *C. albicans* pathogenesis, and transcription of *SOD5* was increased in hyphal growth (Naglik et al., 2003; Sorgo et al., 2010; Sorgo et al., 2011; Röhm et al., 2013; Okamoto-Shibayama et al., 2014). Similarly, we showed that *S. aureus*-promoted *C. albicans* secreted higher levels of Csa2, Rbt4, Sap4-6, and Sod5. Deletion of *RBT4* (Naglik et al., 2003; Martchenko et al., 2004; Sorgo et al., 2010; Sorgo et al., 2011; Röhm et al., 2013; Okamoto-Shibayama et al., 2014), *SOD5* (Martchenko et al., 2004), *SAP4*, and *SAP6 (* Braun et al., 2000) significantly attenuates or completely diminishes *C. albicans* lethality in animal models, partially due to the depletion of *RBT4*-mediated resistance to leucocyte attack (Naglik et al., 2003; Martchenko et al., 2004; Sorgo et al., 2010; Sorgo et al., 2011; Röhm et al., 2013; Okamoto-Shibayama et al., 2014), *SAP4-6*-mediated resistance to macrophage killing (Borg-von Zepelin et al., 1998), and *SOD*-aided protection against intracellular neutrophil killing (Martchenko et al., 2004). In addition to these proteins, we found that hyphae-related proteins (Bailey et al., 1996; Martin et al., 2013), such as Als1, Als3, Ihd1, and Hyr1, were higher in co-cultures over monoculture. This is in agreement with microscopic/morphological characterization, showing increased hyphae in co-culture (data not shown). Furthermore, the co-culture promotes the level of Xog1 in the secretome. Xog1 is an exo-1,3-beta-glucanase essential for reducing beta-glucan epitope exposure (β-glucan masking) to immune cells, hence reducing the phagocytotic interaction and enhancing immune evasion (Ballou et al., 2016; Childers et al., 2020). Two other cell wall crosslinking enzymes Crh11 and Utr2 were also upregulated during co-culturing. Crh11 and Utr2 were shown to impact *β*-glucan masking mildly (Ballou et al., 2016). Together, these results support the contribution of these proteins in *C. albicans–S. aureus* co-culture to the pathogenesis during coinfection.

### *S. aureus* virulence is significantly promoted by *C. albicans* during co-culturing

Similarly to *C. albicans*, the co-culturing also promotes the virulence potential of *S. aureus* by increasing the secretary level of cytolytic, proteolytic, or lipolytic proteins. We found that *C. albicans-*mediated pH maintenance is a critical factor in regulating these proteins. This is consistent with previous observations, where *C. albicans* tended to maintain a neutral pH during co-culturing (**Supplementary Figure S1A**) (Todd et al., 2019b; Pasman et al., 2024) and, thereby, promoted the production and secretion of alpha hemolysin (hla) (Todd et al., 2019b), and hlb and hlg. This is likely due to the *C. albicans*-mediated activation of the P3 promoter of the *S. aureus agr* system, which increases the expression of RNAIII that is essential for the production and secretion of these hemolysing toxins (Novick et al., 1993; Dunman et al., 2001). While we and others confirmed the contribution of pH in regulating *S. aureus* virulence factors, other unknown *C. albicans*-derived factors also contributed to the elevated virulence potential of *S. aureus*. For example, the ECVFs were significantly decreased in buffered monocultures over unbuffered monoculture but significantly increased in co-culture over unbuffered monoculture. These results further highlighted the nuanced regulation of the secreted virulence factors and further studies are warranted to identify these *C. albicans-*derived factors.

As many virulence factors from both *C. albicans* and *S. aureus* were substantially increased in co-culturing, it is not surprising that the secretome from co-culture exhibited higher cytotoxicity toward Ca 9–22 and HO1N1 cells compared with that from the monocultures. Consistently, *C. albicans–S. aureus* co-culturing showed higher cytotoxicity toward keratinocytes NOK-si and HaCat cells, albeit to a different extent (de Carvalho Dias et al., 2017). This discrepancy might be due to the different sensitivity of these cell lines.

### *C. albicans* and *S. aureus* reciprocally promote the iron acquisition potential of each other

An important strategy for pathogenic microbes is to exploit host heme/iron sources during the infection. The increase in both *C. albicans* and *S. aureus* iron acquisition potential may also explain the high lethality of co-infection *in vivo* in animal models (Carlson, 1983). For *C. albicans*, Csa2 is critical in the uptake of hemoglobin and heme proteins and their utilization as an iron source (Okamoto-Shibayama et al., 2014) and this process is assisted by Rbt5, Pga7, Frp1, and Frp2 (Kuznets et al., 2014; Nasser et al., 2016; Roy et al., 2022). We found that co-culturing increases the secreted level of Csa2, but not Rbt5, Pga7, Frp1, and Frp2 in *C. albicans*. For *S. aureus*, the Isd (iron-regulated surface determinant) system is essential for hemoglobin and heme binding, uptake, and iron release (Caza and Kronstad, 2013). Of all the isd proteins, the levels of cell wall anchored isdA, isdB, isdC, isdE, and isdH, but not intracellular isdI and isdG, were significantly higher in co-culture, supporting the potential contribution of these proteins in the *in vivo* pathogenesis. Nonetheless, a previous study found that none of isd proteins was significantly changed in co-culture over monoculture (Peters et al., 2010).

### Limitations of the study

While we systemically elucidated the reciprocal effects of *C. albicans* and *S. aureus* on their virulence factor secretion, cytotoxicity, and proinflammatory effects, there are several limitations of this study. First, the co-culturing conditions did not perfectly match the *in vivo* microenvironment, despite our effort to develop a completely defined medium to remove unknown artificial co-founding factors in the culturing media (Pasman et al., 2024). Future investigation may consider more physiologically relevant *in vitro* systems such as the reconstructed human gingiva–microbe interaction model (Zhang et al., 2022). In addition, all the culturing was performed under an oxygenated environment. Similar to our study, most studies carried out *in vitro C. albicans* cultures in a regular CO_2_ incubator that maintains 5% CO_2_. These conditions do not necessarily represent the *in vivo* physiology, since commensal *C. albicans* cells reside in the oral cavity and large intestine, where the oxygen tension can be relatively low. Furthermore, it must be noted that lowered biofilm DNA concentrations were observed regarding the *C. albicans ALS1/ALS3* ΔΔ/ΔΔ conditions with respect to its wild-type strain (**Supplementary Figure S1**). However, no differences were observed between C. albicans wild-type and *ALS1/ALS3* ΔΔ/ΔΔ in secretome protein concentrations (**Supplementary Figure S1**). Finally, we used THP-1-derived macrophages instead of primary macrophages or primary monocyte-derived macrophages. Despite that THP-1 cells are widely used to model human monocytes and macrophages (Mohd Yasin et al., 2022), it has been shown that THP-1 macrophages may show different response patterns as the primary macrophages (Hoppenbrouwers et al., 2022).

## Data Availability

The datasets presented in this study are deposited in the Gene Expression Omnibus repository, accession number GSE289787; the proteomexchange repository, accession number PXD061998.
