# Supplementary material for: Candida albicans and Staphylococcus aureus reciprocally promote their virulence factor secretion and pro-inflammatory effects
Source: Front Cell Infect Microbiol. 2025 Aug 22;15:1629373. doi: 10.3389/fcimb.2025.1629373 (PMC12411458; doi:10.3389/fcimb.2025.1629373)
Supplement: Supplementary file 1 [file SupplementaryFile1.docx]

**Supplementary information**

***Candida albicans* and *Staphylococcus aureus* reciprocally promote their virulence factor secretion and pro-inflammatory effects**

**Raymond Pasman,^1^ Bastiaan P. Krom,^2^ Martijs J. Jonker,^3^ Wim C. de Leeuw,^3^ Gertjan Kramer,^4^ Stanley Brul,^1^ Sebastian A.J. Zaat,^5^ and Jianbo Zhang^1, 6, *^**

^1^Department of Molecular Biology and Microbial Food Safety, Swammerdam Institute for Life Sciences, University of Amsterdam, Amsterdam, The Netherlands

^2^Department of Preventive Dentistry, Academic Centre for Dentistry Amsterdam (ACTA), University of Amsterdam and Free University Amsterdam, Amsterdam, The Netherlands

^3^RNA Biology Research Group, Swammerdam Institute for Life Sciences, University of Amsterdam, Amsterdam, the Netherlands

^4^Laboratory for Mass Spectrometry of Biomolecules, Swammerdam Institute for Life Sciences, University of Amsterdam, Science Park 904, 1098 XH Amsterdam, The Netherlands

^5^Department of Medical Microbiology and Infection Prevention, Amsterdam Institute for Immunology and Infectious Diseases, Amsterdam UMC, University of Amsterdam, Amsterdam, The Netherlands

^6^Tytgat Institute for Liver and Intestinal Research, Amsterdam Gastroenterology, Endocrinology and Metabolism, Amsterdam UMC, Location Academic Medical Center, Amsterdam, 1105 BK the Netherlands

**
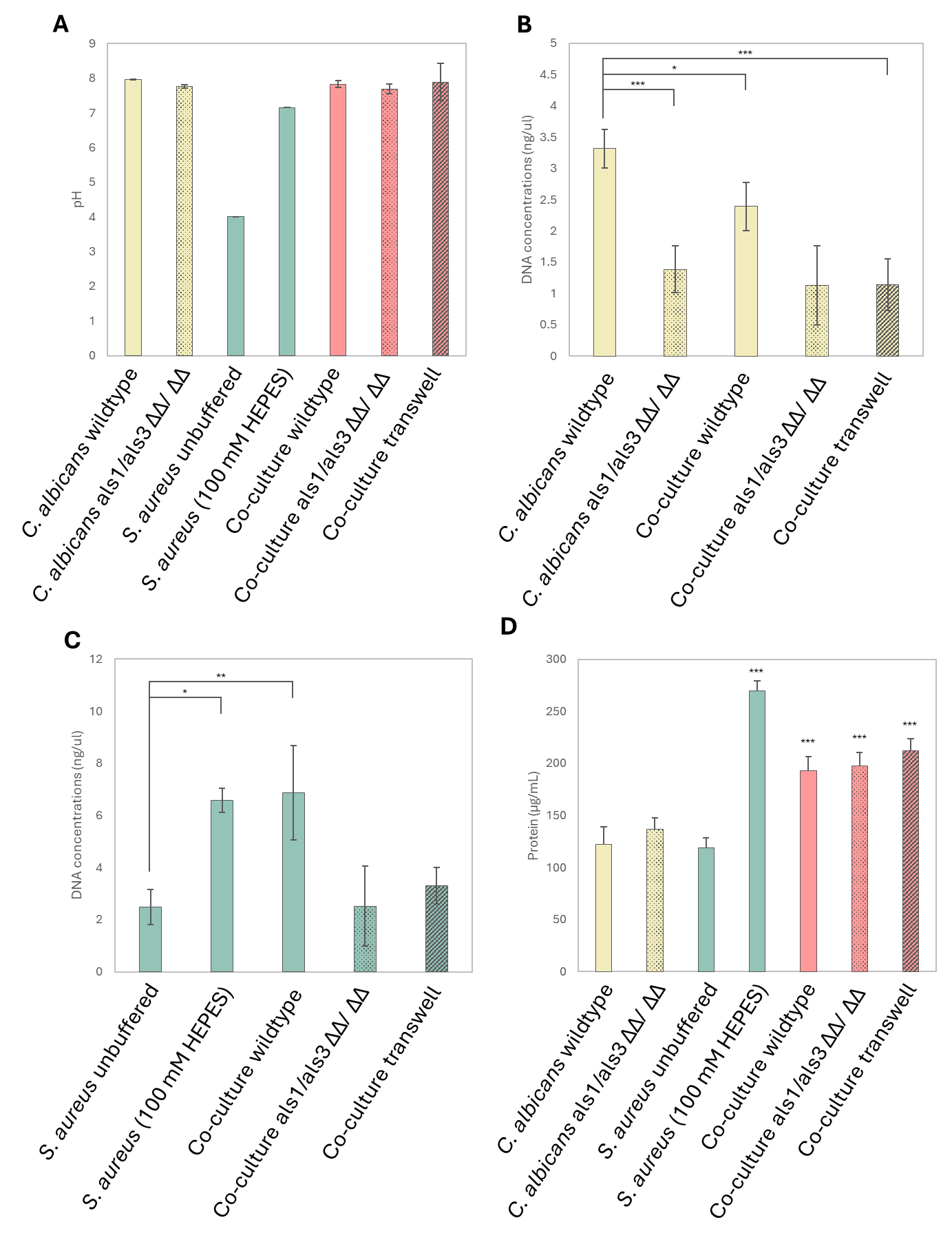
**

**Figure S1. Characterization of media and coculture.** (A) pH values of the media under different culturing conditions. (B-C) Fungal (B) and bacterial (C) DNA concentrations of the coculture. (D) Protein concentration of the concentrated secretome.

**
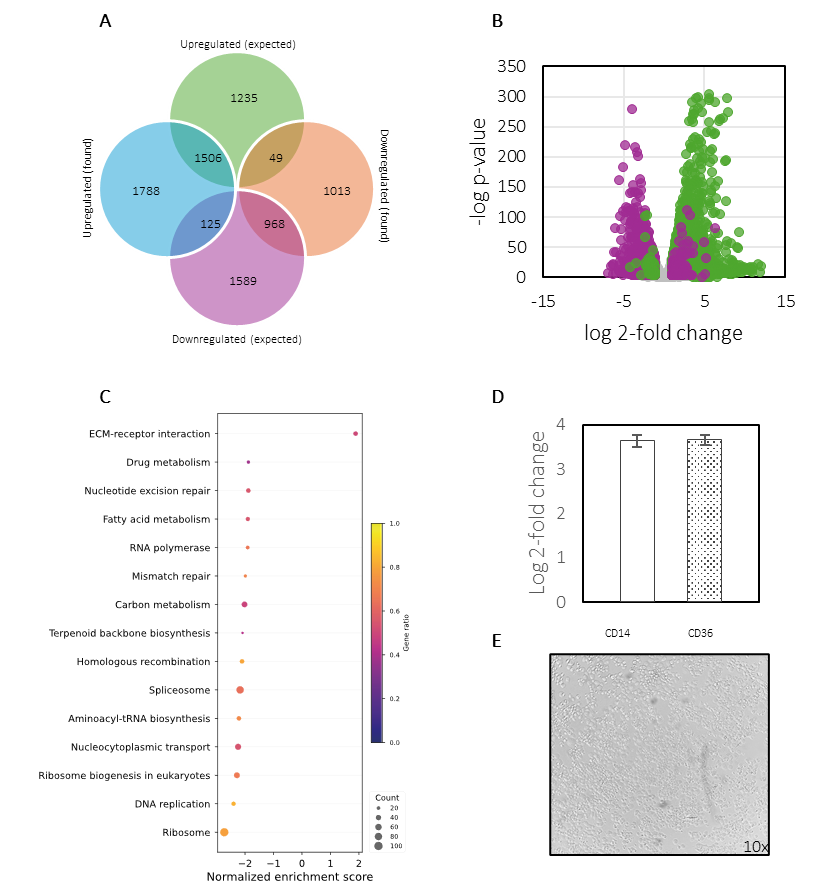
**

**Figure S2. THP-1 monocytes underwent proper differentiation into macrophages at transcriptional level.** (A) Venn diagram comparing expected with found upregulated and downregulated DEGs in PMA-differentiated THP-1 cells compared to undifferentiated THP-1 monocytes. (B) Volcano plot of experimentally found genes overlapping with previously found DEGs, coloured by previously identified upregulated (green) and downregulated (purple) DEGs. Previously identified DEGs that were not differentially regulated in our study are coloured in grey. (C) GSEA performed on Log2 fold changes of differentiated monocytes compared to undifferentiated monocytes with size depicting the total count of genes in each enriched pathway and colour depicting the ratio of found genes compared to the total amount of genes in the corresponding pathway. (D) Log2 fold change of genes *CD14* and *CD36* in differentiated THP-1 macrophages versus undifferentiated THP-1 monocytes. (E) Bright-field image of THP-1 macrophages following 72-hour exposure of PMA.


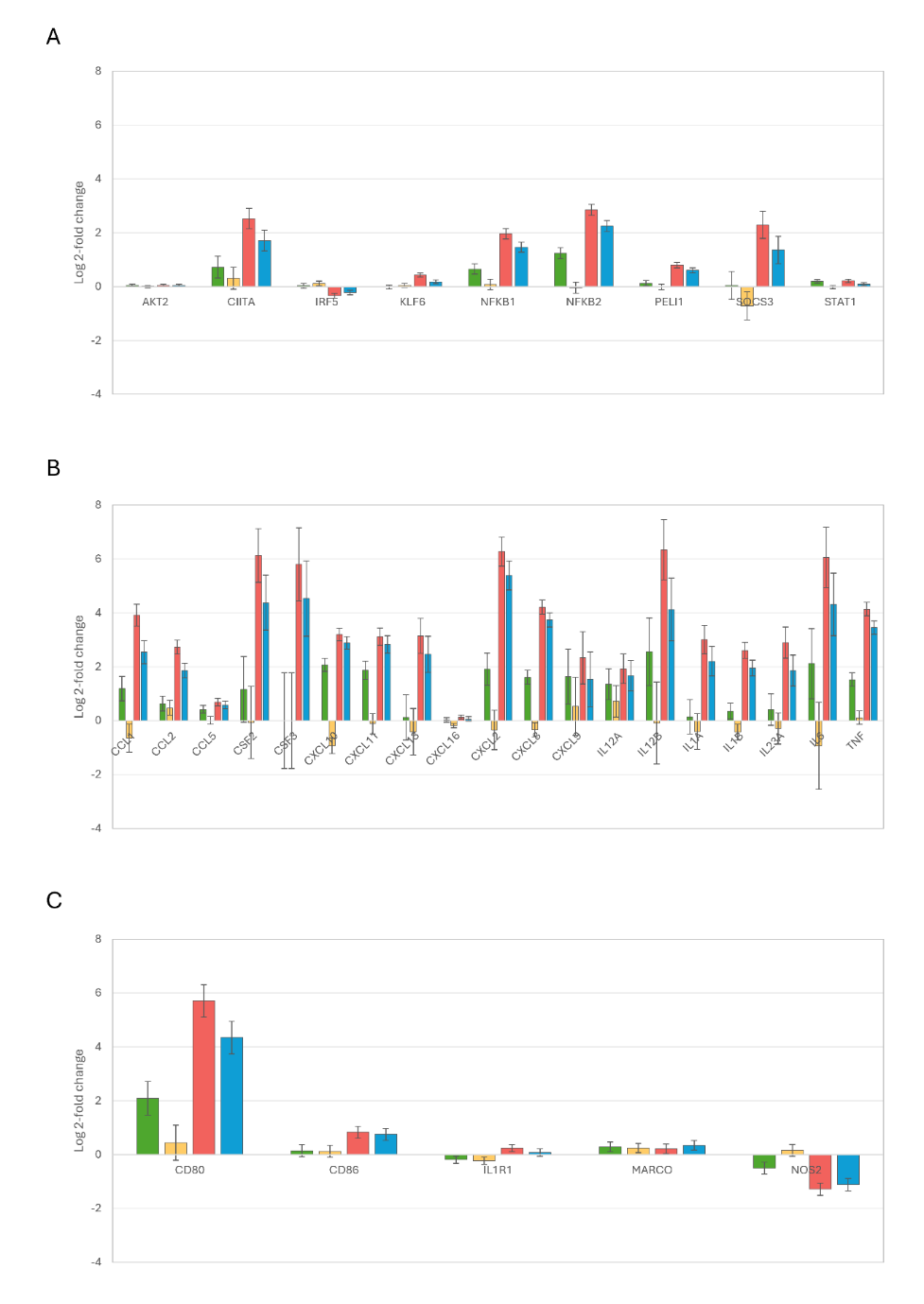


**Figure S3. *S. aureus* secretome promotes expression of M1 polarization related genes in THP-1 M0 macrophages and is increased during co-culturing.** Log2 fold change in expression of genes related to (A) regulation of M1 polarization, (B) M1 cytokines and chemokines, and (C) M1 receptors and enzymes, according to the gene sets for differential gene expression, reported by Rynikova *et al.* (2023) (36). Differences in expression are displayed as the Log2 fold change between unexposed macrophages and macrophages exposed to either *S. aureus* (green), *C. albicans* (yellow), wildtype coculture (red), or *ALS1/ALS3* ΔΔ/ΔΔ coculture (blue) secretomes.


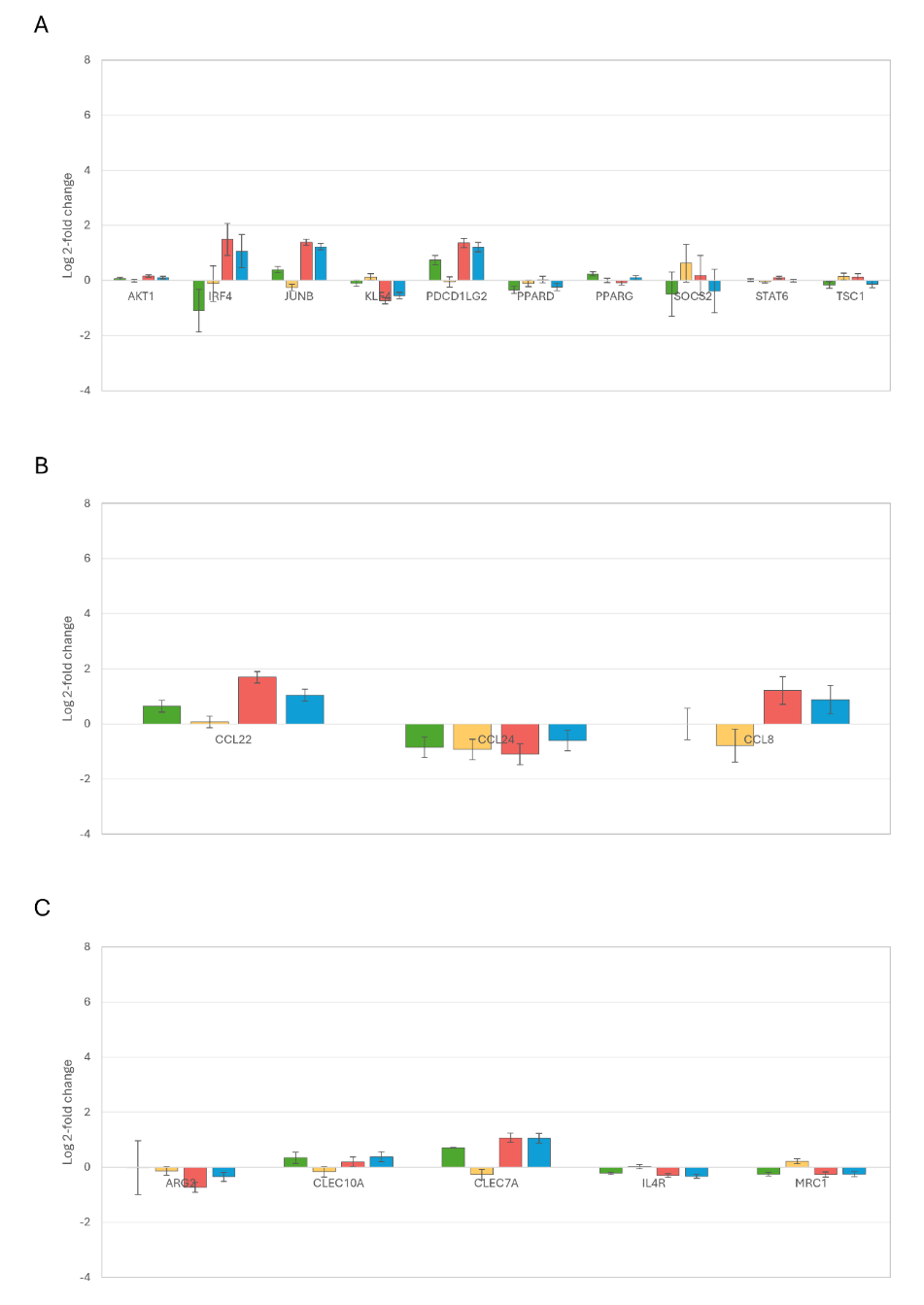


**Figure S4. M2 polarization related genes in THP-1 M0 macrophages are hardly affected by mono and co-culture secretome exposure.** Log2 fold change in expression of genes related to (A) regulation of M2 polarization, (B) M2 cytokines and chemokines, and (C) M2 receptors and enzymes, according to the gene sets for differential gene expression, reported by Rynikova *et al.* (2023) (36). Log2 fold change is displayed as difference between macrophages exposed to either *S. aureus* (green), *C. albicans* (yellow), wildtype coculture (red), or *ALS1/ALS3* ΔΔ/ΔΔ coculture (blue) secretomes over negative control (macrophages in RPMI).

**Table S1**. ***C. albicans* ECVFs are increased by the presence of *S. aureus*.** Significantly differing *C. albicans* ECVF proteins in the secretome of wildtype, trans well, and *ALS1/ALS3* ΔΔ/ΔΔ cocultures. All separated by regulation together with corresponding virulence class, protein description, gene, and log 2-fold change per coculture condition.

| **Regulation** | | **Virulence class** | **Description** | **Protein** | **Log 2-fold change** | | |
| --- | --- | --- | --- | --- | --- | --- | --- |
|  | | | | | **Coculture**  **wildtype** | **Coculture**  ***ALS1/ALS3***  **ΔΔ/ΔΔ** | **Coculture**  **trans well** |
| **Regulated by the presence of *S. aureus*** | | | |  |  |  |  |
| up | Cell wall remodelling | | Glycosidase | Crh11 | 2,94 | 0,35 | 1,68 |
| up | Enzyme (proteolytic) | | Secreted aspartic protease 5 | Sap5 | 2,72 | 1,01 | 1,46 |
| **Regulated by Als1p/Als3p binding to soluble *S. aureus* factors** | | | | |  |  |  |
| up | Cell wall remodelling | | Glycosidase | Utr2 | 2,93 | ns | 1,40 |
| up | Enzyme (proteolytic) | | Secreted aspartic protease 9 | Sap9 | 1,85 | ns | 0,94 |
| up | Immune evasion | | PR-1 proteins homologue | Rbt4 | 2,91 | -0,23 | 1,11 |
| up | Immune evasion | | PR-1 proteins homologue | Rbe1 | 1,71 | -1,63 | 1,67 |
| up | Immune evasion | | Glucan 1,3-beta-glucosidase | Xog1 | 1,40 | -2,00 | 0,82 |
| **Regulated by physical binding of Als1p/Als3p to *S. aureus*** | | | | |  |  |  |
| up | Enzyme (lipolytic) | | phospholipase 1 | Plb1 | 2,16 | ns | ns |
| up | Enzyme (proteolytic) | | Secreted aspartic protease 4 | Sap4 | 1,85 | -0,90 | -0,66 |
| up | Enzyme (proteolytic) | | Secreted aspartic protease 6 | Sap6 | 2,45 | -0,51 | -0,17 |
| up | Immune evasion | | Superoxide dismutase 5 | Sod5 | 1,73 | -1,60 | ns |
| up | Iron utilization | | Secreted hemophore | Csa2 | 2,18 | -1,42 | ns |
| **Not detected** | | | |  |  |  |  |
|  | Enzyme (proteolytic) | | Secreted aspartic protease 1 | Sap1 |  |  |  |
|  | Enzyme (proteolytic) | | Secreted aspartic protease 2 | Sap2 |  |  |  |
|  | Enzyme (proteolytic) | | Secreted aspartic protease 3 | Sap3 |  |  |  |
|  | Enzyme (proteolytic) | | Secreted aspartic protease 7 | Sap7 |  |  |  |
|  | Enzyme (proteolytic) | | Secreted aspartic protease 8 | Sap8 |  |  |  |
|  | Enzyme (proteolytic) | | Secreted aspartic protease 10 | Sap10 |  |  |  |
|  | Cell wall remodelling | | Glycosidase | Crh12 |  |  |  |

**Table S2. *C. albicans* Non-extracellular virulence factors found in medium under different conditions**

| **Regulation** | **Virulence class** | **Description** | **Gene** | **Log 2-fold change** | | | |
| --- | --- | --- | --- | --- | --- | --- | --- |
| **Regulated by the presence of *S. aureus*** | | |  | **Coculture**  **wildtype** | **Coculture**  ***ALS1/ALS3* ΔΔ/ΔΔ** | **Coculture**  **Transwell** |  |
| up | Immune evasion | Hyphae regulated cell wall protein 1 | Hyr1 | 2,44 | 1,48 | 1,78 |  |
| up | Adhesion | Heat shock protein 70 | Ssa1 | 2,41 | 1,29 | 0,43 |  |
| down | Immune evasion | Thioredoxin peroxidase | Ahp1 | -1,25 | -2,57 | -0,93 |  |
| down | Adhesion | Glycolipid 2-alpha-mannosyltransferase 1 | Mnt1 | -1,06 | -2,32 | -0,88 |  |
| **Regulated by Als1p/Als3p binding to soluble *S. aureus* factors** | | |  |  |  |  |  |
| up | Immune evasion | Cell wall protein | Rhd3 | 2,56 | -1,07 | 1,38 |  |
| up | Cell wall remodelling | Induced during hyphae development protein 1 | Ihd1 | 2,76 | -0,52 | 0,56 |  |
| up | Cell wall remodelling | Beta-glucosidase | Sun41 | 2,87 | ns | 1,00 |  |
| up | Cell wall remodelling | 1,3-beta-glucanosyltransferase | Pga4 | 2,90 | -0,37 | 1,07 |  |
| up | Adhesion | Agglutinin-like sequence protein 3 | Als3 | 0,55 | -0,43 | 0,19 |  |
| up | Adhesion | Agglutinin-like sequence protein 1 | Als1 | 0,82 | ns | 0,49 |  |
| up | Adhesion | Cell surface mannoprotein | Mp65 | 2,95 | ns | 1,20 |  |
| up | Cell wall remodelling | Glucan 1,3-beta-glucosidase | Bgl2 | 2,76 | ns | 1,64 |  |
| **Regulated by physical binding of Als1p/Als3p to *S. aureus*** | | |  |  |  |  |  |
| up | Immune evasion | Glutathione reductase | Glr1 | 1,17 | -2,20 | -0,75 |  |
| **Regulated differently** | | |  |  |  |  |  |
| **••** | Enzyme | Enolase | Eno1 | ns | -1,82 | -1,64 |  |

**Table S3**. ***S. aureus* ECVFs are mainly increased by the presence of *C. albicans* and its pH maintenance.**  Significantly differing *S. aureus* ECVF proteins in the secretome of wildtype, trans well, and *ALS1/ALS3* ΔΔ/ΔΔ cocultures as well as buffered monocultures. All separated by regulation together with corresponding virulence class, protein description, gene, and log 2-fold change per culture condition.

| **Regulation** | | **Virulence class** | **Description** | **Gene** | **Log 2-fold change** | | | |
| --- | --- | --- | --- | --- | --- | --- | --- | --- |
|  | | | | | **Coculture**  **wildtype** | **Coculture**  ***ALS1/ALS3* ΔΔ/ΔΔ** | **Coculture**  **trans well** | ***S. aureus***  **(buffered)** |
| **Regulated by *C. albicans* pH control** | | | | |  |  |  |  |
| up | Toxin (cytolytic) | | Alpha hemolysin | hly/hla | 2,14 | 2,02 | 2,41 | 0,72 |
| up | Toxin (cytolytic) | | Beta hemolysin | hlb | 2,29 | 2,23 | 2,34 | 1,11 |
| up | Toxin (cytolytic) | | Gamma hemolysin subunit A | hlgA | 2,16 | 2,29 | 0,39 | 0,84 |
| up | Toxin (cytolytic) | | Gamma hemolysin subunit B | hlgB | 2,34 | 2,24 | 2,14 | 0,80 |
| up | Toxin (cytolytic) | | Gamma hemolysin subunit C | hlgC | 2,34 | 2,18 | 1,91 | 0,66 |
| **Regulated by the presence of *C. albicans*** | | | | |  |  |  |  |
| up | Enzyme (proteolytic) | | Cysteine proteinase staphopain B | sspB | 0,49 | 0,61 | 1,00 | -1,68 |
| up | Enzyme (proteolytic) | | Serine protease-like protein A | splA | 1,51 | 1,52 | 1,93 | -0,44 |
| up | Enzyme (proteolytic) | | Serine protease-like protein B | splB | 1,01 | 0,95 | 1,25 | -1,28 |
| up | Enzyme (proteolytic) | | Serine protease-like protein C | splC | 0,58 | 0,45 | 2,23 | -0,58 |
| up | Enzyme (proteolytic) | | Serine protease-like protein D | splD | 1,53 | 1,45 | 1,68 | ns |
| up | Enzyme (proteolytic) | | Serine protease-like protein E | splE | 1,51 | 1,28 | 1,65 | -0,74 |
| up | Enzyme (proteolytic) | | Serine protease-like protein F | splF | 0,87 | 0,80 | 1,05 | -1,45 |
| up | Enzyme (lipolytic) | | Lipase | lip | 0,53 | 0,63 | 1,28 | -1,50 |
| up | Enzyme (lipolytic) | | Phospholipase C | plc | 0,42 | 0,42 | 1,27 | -1,54 |
| up | Toxin (cytolytic) | | Delta hemolysin | hld | 0,77 | 1,28 | 2,05 | ns |
| up | Toxin (cytolytic) | | Leukotoxin D | lukD | 1,89 | 1,68 | 1,78 | ns |
| **Regulated by physical binding to Als1/Als3** | | | | |  |  |  |  |
| up | Immune evasion | | Staphylococcal superantigen-like 7 | ssl7 | 1.21 | ns | ns | -1.17 |
| **Regulated differently** | | | | |  |  |  |  |
| (down) | Enzyme (proteolytic) | | Cysteine proteinase staphopain A | sspA | -1,08 | -0,81 | ns | -2,57 |
| (down) | Enzyme (lipolytic) | | Triacylglycerol lipase | lip2 | -0,91 | ns | -0,66 | -2,63 |
|  | Enzyme | | Thermo nuclease | nuc | ns | 0,38 | 0,41 | -2,04 |

**Table S4 *S. aureus* non-extracellular virulence factors found in medium under different conditions**

| **Regulation** | **Virulence class** | **Description** | **Gene** | **Log 2-fold change** | | | |
| --- | --- | --- | --- | --- | --- | --- | --- |
| **Regulated by *C. albicans* pH control** | | |  | **Coculture**  **wildtype** | **Coculture**  ***ALS1/ALS3ΔΔ/ΔΔ*** | **Coculture**  **trans well** | ***S. aureus***  **(buffered)** |
| up | Immune evasion | d-alanine-d-alanyl carrier protein ligase | dlta | 2,65 | 2,31 | 2,06 | 1,44 |
| up | Enzyme | Enolase | eno | 2,51 | 2,24 | 1,80 | 0,93 |
| down | Adhesion | Clumping factor B | clfB | -0,38 | -0,31 | -0,31 | -2,56 |
| down | Adhesion | Elastin binding protein | ebp | -0,61 | -0,68 | -0,80 | -2,71 |
| **Regulated by the presence of *C. albicans*** | | |  |  |  |  |  |
| up | Antioxidant | Alkyl hydroxide reductase | ahpc | 1,92 | 1,89 | 1,53 | ns |
| up | Enzyme | O-acetyltransferase | oata | 0,58 | 0,69 | 0,45 | -1,80 |
| up | Adhesion | Serine-rich surface protein | srap/sasa | 0,56 | 0,66 | 0,83 | -1,72 |
| up | Iron acquisition | Iron-regulated surface determinant B | isdB | 0,49 | 0,46 | 0,20 | -2,00 |
| up | Iron acquisition | Iron-regulated surface determinant E | isdE | 1,63 | 1,56 | 1,10 | -0,72 |
| up | Iron acquisition | Iron-regulated surface determinant H | isdH | 2,17 | 1,87 | 1,65 | ns |
| **Regulated by biofilm integration** | | |  |  |  |  |  |
| up | Iron acquisition | Iron-regulated surface determinant A | isdA | 0,49 | 0,46 | ns | -2,00 |
| up | Iron acquisition | Iron-regulated surface determinant C | isdC | 0,94 | 0,74 | ns | -1,71 |
| **Regulated by binding to soluble Als1p/Als3** | | |  |  |  |  |  |
| up | Adhesion | Ser-Asp rich fibrinogen-binding protein | sdrD | 0,30 | ns | 0,36 | ns |
| **Regulated by physical binding to Als1p/Als3p** | | |  |  |  |  |  |
| up | Adhesion | Extracellular adherence protein/  MHC analogous protein | eap/map | 2.04 | ns | ns | ns |
| **Regulated differently** | | |  |  |  |  |  |
|  | Adhesion | Staphylococcal protein A | spA | -0,34 | -0,45 | ns | -2,56 |
|  | Adhesion | Ser-Asp rich fibrinogen-binding proteins | sdrC | ns | -0,19 | ns | -2,41 |
|  | Adhesion | Clumping factor A | clfA | ns | ns | ns | -2,34 |
